# Selecting, refining and identifying priority Cochrane Reviews in health communication and participation in partnership with consumers and other stakeholders

**DOI:** 10.1186/s12961-019-0444-z

**Published:** 2019-04-29

**Authors:** Anneliese J. Synnot, Allison Tong, Peter Bragge, Dianne Lowe, Jack S. Nunn, Molly O’Sullivan, Lidia Horvat, Debra Kay, Davina Ghersi, Steve McDonald, Naomi Poole, Noni Bourke, Natasha A. Lannin, Danny Vadasz, Sandy Oliver, Karen Carey, Sophie J. Hill

**Affiliations:** 1 Centre for Health Communication and Participation, School of Psychology and Public Health, La Trobe University, Melbourne, Australia; 2 Cochrane Australia, School of Public Health and Preventive Medicine, Monash University, Melbourne, Australia; 3 Sydney School of Public Health, The University of Sydney, Sydney, Australia; 4 Centre for Kidney Research, The Children’s Hospital at Westmead, Sydney, Australia; 5 BehaviourWorks Australia, Monash Sustainable Development Institute, Monash University, Melbourne, Australia; 6 Murdoch Children’s Research Institute, Melbourne, Australia; 7 Safer Care Victoria, Melbourne, Australia; 8 South Australian Health and Medical Research Institute, Adelaide, Australia; 9 NHMRC Clinical Trials Centre, Sydney Medical School, The University of Sydney, Sydney, NSW Australia; 10 National Health and Medical Research Council, Canberra, Australia; 11 Australian Commission on Safety and Quality in Healthcare, Sydney, Australia; 12 Bass Coast Health, Wonthaggi, Australia; 13 Alfred Health, Melbourne, Australia; 14 School of Allied Health (Occupational Therapy), La Trobe University, Melbourne, Australia; 15 Health Issues Centre, Melbourne, Australia; 16 University College London, London, United Kingdom; 17 University of Johannesburg, Johannesburg, South Africa

**Keywords:** Health communication, patient participation, health priorities, community participation, patient-centred care, decision-making

## Abstract

**Background:**

Priority-setting partnerships between researchers and stakeholders (meaning consumers, health professionals and health decision-makers) may improve research relevance and value. The Cochrane Consumers and Communication Group (CCCG) publishes systematic reviews in ‘health communication and participation’, which includes concepts such as shared decision-making, patient-centred care and health literacy. We aimed to select and refine priority topics for systematic reviews in health communication and participation, and use these to identify five priority CCCG Cochrane Reviews.

**Methods:**

Twenty-eight participants (14 consumers, 14 health professionals/decision-makers) attended a 1-day workshop in Australia. Using large-group activities and voting, participants discussed, revised and then selected 12 priority topics from a list of 21 previously identified topics. In mixed small groups, participants refined these topics, exploring underlying problems, who they affect and potential solutions. Thematic analysis identified cross-cutting themes, in addition to key populations and potential interventions for future Cochrane Reviews. We mapped these against CCCG’s existing review portfolio to identify five priority reviews.

**Results:**

Priority topics included poor understanding and implementation of patient-centred care by health services, the fact that health information can be a low priority for health professionals, communication and coordination breakdowns in health services, and inadequate consumer involvement in health service design. The four themes underpinning the topics were culture and organisational structures, health professional attitudes and assumptions, inconsistent experiences of care, and lack of shared understanding in the sector. Key populations for future reviews were described in terms of social health characteristics (e.g. people from indigenous or culturally and linguistically diverse backgrounds, elderly people, and people experiencing socioeconomic disadvantage) more than individual health characteristics. Potential interventions included health professional education, interventions to change health service/health professional culture and attitudes, and health service policies and standards. The resulting five priority Cochrane Reviews identified were improving end-of-life care communication, patient/family involvement in patient safety, improving future doctors’ communication skills, consumer engagement strategies, and promoting patient-centred care.

**Conclusions:**

Stakeholders identified priority topics for systematic reviews associated with structural and cultural challenges underlying health communication and participation, and were concerned that issues of equity be addressed. Priority-setting with stakeholders presents opportunities and challenges for review producers.

**Electronic supplementary material:**

The online version of this article (10.1186/s12961-019-0444-z) contains supplementary material, which is available to authorized users.

## Background

Historically, the health research agenda has largely been set by researchers and funders with little input from the end users of research [1], including consumers (patients and their families or carers, and the general public) [2] and other stakeholders (health professionals, health policy-makers and decision-makers). This has been shown to result in a mismatch between the priorities of the users of research and the research that is conducted [3, 4]. This mismatch can be a driver of research waste [3], and may mean that health research fails its most fundamental objective, that is to improve health and treatment outcomes. Priority-setting partnerships between the research community and consumers and other stakeholders are gaining popularity as a mechanism to ensure often limited public funds are directed to research that better meets the needs of those whose lives the research affects as well as the expectations of the broader community [5, 6].

These moves towards partnerships in health research reflect broader health system shifts. Health funders and providers around the world seek to deliver health systems that are person centred, where people receive safe, timely and culturally appropriate care, and can make informed decisions about their health in partnership with their health professionals [7–9]. To support this, consumers should be partners at all levels of healthcare, from individual care to health system planning and governance [9]. An important input into decisions about improving health systems and services is evidence from relevant research [10]. Systematic reviews, as summaries of multiple studies on a topic, are an appropriate and reliable source of evidence to inform health decision-making [11].

Cochrane is a global, independent, not-for-profit organisation with an international network of contributors who conduct and publish systematic reviews (termed Cochrane Reviews). To ensure the relevance of their reviews to health decision-making, Cochrane recently adopted strategic objectives related to the prioritisation of Cochrane Reviews [12] and a number of Cochrane groups have undertaken comprehensive priority-setting activities with stakeholders specific to their topic scope [13–17]. Importantly, considerable guidance exists to generate broadly scoped research priorities [18–20], but the methods and ‘real-world’ considerations to inform the subsequent formulation and selection of answerable systematic review questions are still developing [21, 22].

Within Cochrane, the Cochrane Consumers and Communication Group (CCCG) is responsible for coordinating the publication of systematic reviews of “*interventions that affect the way people interact with healthcare professionals, services and researchers*” [23]. Described in this project as ‘health communication and participation’, this includes concepts such as shared decision-making, person-centred care, patient experience-led improvement, health literacy, and the co-design of health services, policy and research. Many of these concepts have already been identified as research priorities for consumers and other stakeholders in diverse clinical areas such as intensive care [24], kidney disease [14] and asthma [25]. At project commencement, we could find no information on stakeholder-generated research priorities across the broad scope of health communication and participation that CCCG could use to help prioritise their systematic review portfolio. As a result, in 2015–2016, CCCG undertook a comprehensive research priority-setting activity with consumers and other stakeholders to identify future Cochrane Reviews in health communication and participation. In the first stage, reported elsewhere [26], we used an international online survey to identify the broad research priorities of stakeholders in this area. The aims of the second stage, reported here, were to select and refine priority topics for systematic reviews in health communication and participation, and from these, to identify five priority CCCG Cochrane Reviews.

## Methods

**Fig 1:**
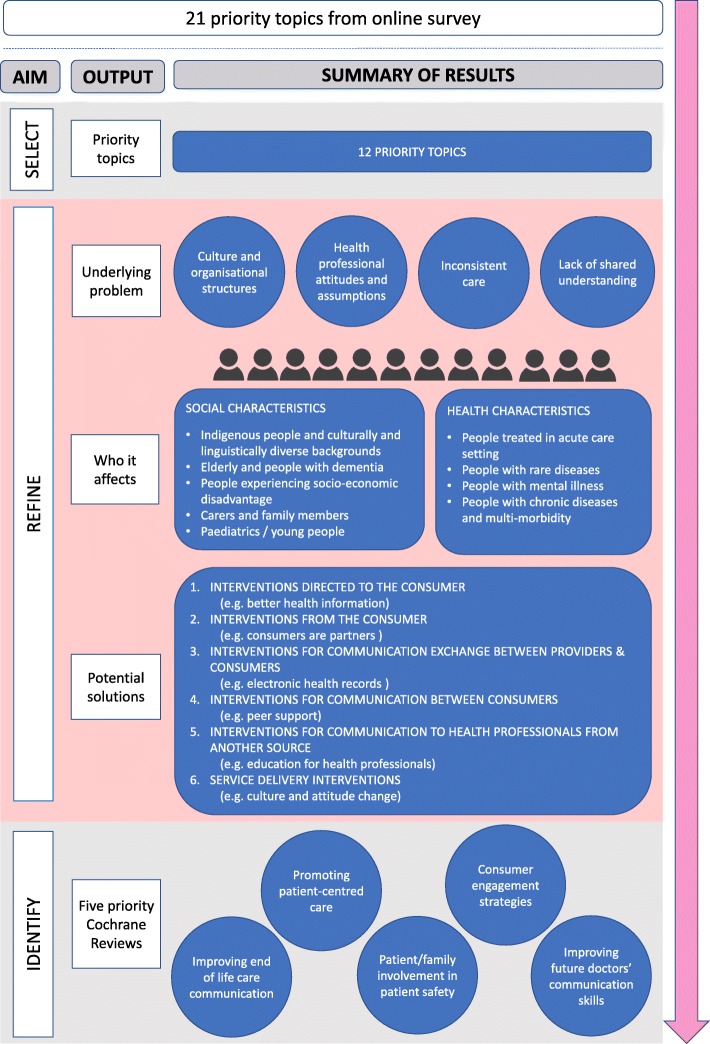
Summary of project aims, corresponding intended outputs and summary of main results

We conducted a workshop with stakeholders to select and refine their priority topics for systematic reviews in health communication and participation. Following this, we mapped their topics against the existing CCCG review portfolio to identify five priority Cochrane Reviews. For a visual summary showing the three aims and their corresponding intended outputs, see Fig. 1.

Underpinning our method was a commitment to work in partnership with consumers and other stakeholders to plan, undertake and disseminate the project [27]. This commitment was informed by the principles of co-production. This includes sharing of power and responsibility, including all perspectives and skills, respecting and valuing contributors’ knowledge, and cultivating mutually beneficial and supportive relationships [28]. Our reporting here is informed by a 32-item priority-setting appraisal checklist [29].

### Context

The project was initiated by researchers at the Centre for Health Communication and Participation (the Centre) in Melbourne, Australia, where CCCG is located [26]. We convened an 11-member steering group representing the following stakeholders: consumers (DK, KC), consumer groups (DV), health policy-makers (LH, NP), health professionals (NL), health services (NB), and research funders (DG). We also included two researchers (PB, SO) with priority-setting expertise for methodological advice, and a representative from Cochrane Australia (SM).

We considered the project to be international in scope (reflecting Cochrane’s global focus) but, for feasibility, we conducted the workshop component (reported here) in Australia. For clarity, we described the topic scope (interventions for health communication and participation) in project materials as “*activities that help patients, consumers and carers to be knowledgeable about their health and to participate in their health in different ways. This includes being able to express their views and beliefs, make informed choices, and access high quality health information and health services*” [30]. It included broader participation in health services, policy and research. More detail about project governance and scope, and the earlier project stage, is provided in Synnot et al. [26].

### Methods to select and refine priority topics

We conducted a 1-day workshop with 28 participants using methods informed by James Lind Alliance guidance for research priority-setting partnerships [20] and previous research priority-setting workshops [14, 31]. We took to the workshop 21 previously identified priority topics for research in health communication and participation, generated via an international online survey with 151 consumers, health professionals, policy-makers and researchers [26] (the 21 priority topics are also listed in the workshop pre-reading pack, see Additional file 1). Our priority topics were framed as problem statements, rather than answerable questions. This was a deliberate decision made in the online survey stage (see Synnot [26] for rationale). We elected not to re-frame the priority topics as answerable systematic review questions prior to the workshop, given the vast number of potential systematic review questions that could be generated for each priority topic, and the potential for misinterpretation if researchers undertook this step without stakeholder input.

#### Workshop participants and recruitment

We included consumers, health professionals and health decision-makers aged 18 years and over who had professional or personal experience in health communication and participation. To support the recruitment of participants with diverse perspectives and experiences, we used a sampling frame [32] and devised operational definitions of our participant groups (Table 1). For feasibility and group manageability, our target was 30 participants, with at least 50% consumers to mitigate potential power imbalances and ensure ample inclusion of non-professional perspectives [20]. We used a mix of purposive recruitment via the networks of the Centre and steering group, and snowball recruitment via participants from the earlier online survey. We offered all participants reimbursement for travel-related expenses plus a $50 voucher for those attending outside their paid employment.

**Table 1:** Participant sampling frame and operational definitions used to guide workshop recruitment

Participant group	Operational definition	Target (*n*)	Additional characteristics
Consumer/carer representatives^a^	Works with or represents others with a particular health interest or condition^b^ [2] (e.g. people on health service advisory groups, consumer researchers and peer support workers) [63]	15	Across the participants groups, we sought to include people with the following backgrounds or diversity of experiences: • Indigenous • Culturally and linguistically diverse • Geographic location • Age • Health settings (i.e. community vs. acute care) • Health conditions
Health professionals or health service managers	Has a specific role or interest in health communication and participation (with or without a clinical role), across a mix of professional backgrounds (i.e. doctor, nurse, allied health professional, quality manager or health charity)	10	
Health policy-makers or researchers	Has a specific role in policy or in funding research or services in health communication and participation	5	

^a^We described consumers and carers as separate groups to reflect Australian norms [9] and in light of their potentially different views and perspectives on healthcare provision

^b^We used this definition of ‘consumer and carer representatives’ to ensure we included people who could bring the perspectives and experiences of others, not solely their own lived experience

#### Workshop methods

We held the 6-hour workshop at an accessible, central location in Melbourne, Australia, in September 2015. The facilitation was led by PB, a steering group member with expertise in facilitation and priority-setting, with support from six co-facilitators (AS, SH, DL, JN, LH, SM; all Centre staff or steering group members with experience in focus group facilitation or adult learning). We sent participants a pre-reading pack prior to the workshop (Additional file 1).

Participants selected a place at one of five tables in the room, each with a capacity to seat six. On the day, participants were given hard copies of (1) a brief biography of participants and facilitators, (2) the pre-reading pack, and (3) a more detailed description of the 21 stakeholder-generated priority topics previously identified. The detailed descriptions of these previously identified 21 priority topics were also printed onto individual A3 posters and displayed on the walls (see Additional file 2 for an example).

**Fig 2:**
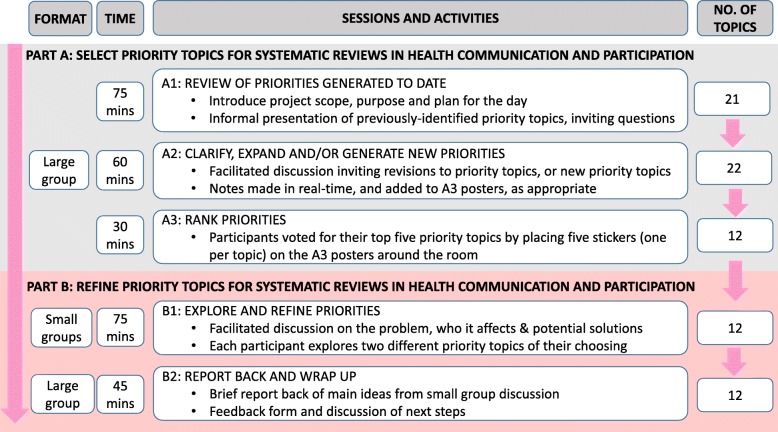
Workshop format, time, sessions and activities, with the corresponding number of priority topics throughout the day

The workshop consisted of five different sessions and used a combination of large and small group activities. We modified the Global Evidence Mapping workshop methods [31] to select priority topics for systematic reviews in health communication and participation (Fig. 2, part A). We then facilitated small group work to refine these priorities further [15, 33], exploring the problem, who it affects and potential solutions (Fig. 2, part B) to inform the identification of future Cochrane Reviews. We took notes during large group activities and audio-recorded and took notes during the small group activities.

##### Workshop part A: selection of priority topics

After general introductions, we delivered a presentation in which we introduced relevant concepts such as systematic reviews, explained how we generated the 21 priority topics (described in the pre-reading pack, Additional file 1) and described each topic in detail (see Fig. 2, session A1, and workshop agenda, Additional file 3). We then invited participants to seek clarification on any aspect of the 21 priority topics and suggest whether anything was missing or to suggest a new topic (Fig. 2, session A2). The lead facilitator typed notes in real-time that were projected onto a screen. Each suggestion was discussed, with co-facilitators proposing whether they thought the suggestion fell within the scope of an existing priority topic or was a new topic of its own. Once this was informally agreed within the room, a co-facilitator amended the A3 posters, as appropriate.

We then moved onto ranking the priority topics by voting (Fig. 2, session A3). Each participant had five stickers to place on their top five priority topics (one sticker per poster), on the understanding that the top 10 would go forward for further discussion in part B.

##### Workshop part B: refining priority topics

We undertook facilitated small group work to further refine the priority topics [15, 33], inviting participants to explore the problem underpinning the priority, who it affects and offer potential solutions (Fig. 2, part B). Their reflections were used to inform the context, justification or background of a Cochrane Review, particularly important for complex reviews [21], and the commonly used population and intervention components of review inclusion criteria [34]. To do this, participants worked in small groups of up to five people, with a co-facilitator guiding the discussion, using a series of prompts (Fig. 2, session B1, and Additional file 4, small group discussion facilitator template). In the final session (Fig. 2, session B2), co-facilitators provided a brief report-back to the group of the main ideas discussed.

### Methods to identify five priority Cochrane Reviews

#### Thematic analysis of workshop discussions

We conducted a thematic analysis of all written text generated about the priority topics to inform the background, context or justification of, and inclusion criteria for, priority Cochrane Reviews. This also allowed us to refine our understanding of the priority topics and to identify any issues or themes relevant for the implementation of future reviews.

We applied the same taxonomy approach [35] that we used in the previous project stage to analyse the online survey data [26]. The written text was predominantly drawn from the co-facilitator notes made in the small group discussions (Fig. 2, session B1) but included any notes made in the earlier large group sessions, and the original text that was on the A3 posters. One researcher (AS) compiled the written text for each of the 12 priority topics and supplemented the written facilitator notes with audio-recordings from each small group session for clarification and to provide illustrative quotes [36].

In an iterative process using Microsoft Excel, we first grouped the text describing each of the 12 priority topics under one of three conceptual codes, namely the problem, the population and the interventions, reflecting the common framework used to devise systematic review questions [34]. For the problem and population conceptual codes, we used an iterative process to develop and apply a series of sub-codes to characterise their various dimensions. For the intervention conceptual code, we applied the categories of the CCCG intervention taxonomy [37] (which groups interventions according to the direction of communication between consumers and healthcare providers) as the sub-codes. We applied consistent sub-codes to all priority topics, and then used these to identify cross-cutting key themes across the 12 priority topics (rather than per priority topic) given their overlapping nature. All sub-codes and key themes were agreed with a second researcher (SH).

#### Mapping priority topics against CCCG review portfolio

As others have noted, turning stakeholder-generated priority topics into answerable, appropriate and feasible systematic questions is an iterative and collaborative process, usually conducted subsequent to any prioritisation activity and one that must inevitably include systematic review authors and editors [21, 22, 38]. We were unable to identify suitable guidance for this step, and therefore we developed an approach based on evidence mapping [31] and standard editorial processes of scope delineation and feasibility.

First, one researcher (AS) compared the 12 priority topics, cross-cutting underlying problem themes, and key populations and potential interventions generated by the workshop participants against the full list of CCCG Cochrane Reviews (including, at the time, 101 titles, protocols and reviews) [39]. We looked for gaps (where new reviews might be proposed) or areas of overlap (where existing reviews might be updated). We then devised and applied the following editorial criteria to potential Cochrane Review topics: (1) the review can be commenced in a timely manner; (2) there are primary studies for inclusion in the review; (3) there is adequate capacity within the author team and the CCCG to undertake/support the review; and (4) the author team is agreeable to formally including consumers and/or other stakeholders in their review planning, conduct and/or dissemination.

A potential list of priority Cochrane Review titles was then reviewed and discussed with the local and international CCCG editorial teams [40] before final approval by the project steering group and the Cochrane Review author teams involved. The CCCG also decided to limit the number of priority Cochrane Reviews to five. This was an editorial decision, reflecting resource considerations, but the CCCG committed to undertaking a second round of priority reviews, drawn from the results of this project, in the future [41].

The final list of five priority Cochrane Reviews was fed back to workshop participants and participants from earlier stages, and made available to all interested parties in a professionally formatted project report [41]. Several workshop participants and steering group members contributed to the report by providing quotes and/or editing content.

## Results

### Participant demographics

Twenty-eight participants took part in the workshop (Table 2). Half of participants (*n* = 14, 50%) identified as a consumer or carer representative, with roles including consumer and community advisory committee members, board members and voluntary office bearers in organisations such as health services, government departments or agencies, not-for-profit organisations and charities. The same number of participants attended in a professional capacity and were employed in metropolitan and regional hospitals, community health services, Victorian and federal government departments and agencies, national patient organisations, and research funding agencies. These people had a mix of clinical, managerial, policy and client-focussed roles.

**Table 2:** Participant characteristics

Characteristic	Consumer/carer^a^ (*n* = 14)	Professional^b^ (*n* = 14)	All (*n* = 28)
Age (years, mean ± SD, range)	56 ± 14 (37 to 85)	44 ± 11 (30 to 61)	51 ± 14 (30 to 85)
Gender (female; *n*, % of total)	11 (79)	13 (93)	24 (86)
Participant ‘perspective’^c^ (*n*)			
Consumer representative	11		11
Carer representative	4		4
Health professional			
Doctor	1	1	2
Allied health professional	1	2	3
Nurse		2	2
Health service manager (non-clinical role)			
Acute/hospital setting		4	4
Community health setting		1	1
Health charity/not-for-profit organisation	1	2	3
Policy-maker (government department or agency)		4	4
Researcher		1	1
Research funder		1	1
Highest education level (*n*, % of total)			
Secondary school	3 (21)	0 (0)	3 (11)
Occupational certificate or diploma	1 (7)	0 (0)	1 (4)
University bachelor’s degree	3 (22)	5 (36)	8 (29)
University post-graduate degree	7 (50)	9 (64)	16 (57)
Aboriginal or Torres Strait Islander (*n*, % of total)	0 (0)	1 (7)	1 (4)
Non-English-speaking background (*n*, % of total)	1 (7)	2 (14)	3 (11)
Area of residence (*n*, % of total)			
Metropolitan	12 (86)	11 (79)	23 (82)
Regional	2 (14)	3 (21)	5 (18)

^a^Included participants who identified as a consumer or carer representative. Three participants were coded to this category as they were primarily recruited for their consumer roles, but also worked as health professionals/health peak body staff

^b^Included participants who identified as a health professional, health service manager, health peak body or not-for-profit organisation employee, policy-maker, researcher or research funder

^c^Several participants in both stakeholder groups nominated more than one ‘perspective’. As such, the total number of participants across ‘perspectives’ is greater than the total number of participants in each stakeholder group

Most participants were female (*n* = 24, 86%) and lived in a metropolitan area (*n* = 23, 82%). The mean age was 51 ± 14 years, with those attending in a professional capacity younger than those who identified as a consumer or carer representative (44 ± 11 vs. 56 ± 14 years, respectively). Both stakeholder groups were highly educated (post-graduate degree holders numbered 50% or greater in both groups); however, the consumer and carer representatives included a broader range of educational levels, with three participants (21%) having completed secondary school only. Across both participant groups, one person (4%) identified as Aboriginal or Torres Strait Islander, and three people (11%) came from a non-English speaking background. We were expecting an additional two female consumer representatives on the day but they did not attend and gave no explanation of why.

### Flow of priority topics through the workshop

The workshop commenced with 21 previously identified priority topics (Fig. 2). During part A of the workshop, participants added more information to several priority topics (for example, clarification of the problem being described, or an additional population group affected) and we created one new priority topic related to transitions in healthcare, bringing the total number of topics to 22. After participants voted, we intended to take the top 10 priority topics onto part B of the workshop. However, the 9th- to 12th-ranked topics each had the same number of votes, so we selected the top 12 priority topics for part B of the workshop.

### Priority topics selected for systematic reviews in health communication and participation

The 12 priority topics that participants most wanted addressed are presented in Table 3. The most highly ranked of these were (1) the term ‘patient-centred care’ is poorly understood and implemented by health services and health professionals; (2) some health professionals do not provide enough information to patients (some do not think it is a priority); (3) breakdowns in communication and coordination of care between and within health services are common; and (4) health services do not properly involve consumers and carers in health service planning and design. The 8th-ranked topic, on communication vulnerabilities associated with transitions, was added at the workshop as the additional priority topic prior to the voting session.

**Table 3:** Top 12 priority topics for health communication and participation research (Adapted from [26])

Health communication and participation research priority topics	Votes (*n*)
Top 12 priority topics	
1. The term patient-centred care is poorly understood and implemented by health services and health professionals	13
2. Some health professionals do not provide enough information to patients (some health professionals do not think it is a priority)	12
3. Breakdowns in communication and coordination of care between and within health services are common	11
4. Health services do not properly involve consumers and carers in health service planning and design	10
5. There is not enough support or understanding about the needs of older people and end-of-life decisions are poorly understood by patients, families and the community	9
6. Consumers and carers do not always know about all the options or services that exist	9
7. The quality and safety of patient care can be compromised by health services (particularly hospitals) not treating patients holistically	9
8. Transitions between health services are a particularly vulnerable communication time	8
9. There are often two-way barriers to adequate communication and participation (e.g. disability of individual plus discomfort of health professional)	7
10. The general public does not always have enough health literacy to navigate the health system and make health decisions	7
11. Consumers and carers are not always able to participate actively in their care	7
12. Some health professionals do not understand or ask patients about their preferences and priorities	7
Priority topics not ranked in the top 12	
13. Patients do not always understand their health problems, treatment options or their rights	5
14. Health professionals do not always provide enough support for patient decision-making	4
15. ‘Official’ health information can be contradictory and hard to understand, both written and online. Consumers and professionals do not know how to find and assess good quality information online	4
16. Informed consent for treatment and research does not always happen	3
17. Cultural safety is not well-embedded in health services	3
18. Health researchers do not adequately involve patients in research, nor share their findings	3
19. Patients often experience information overload and are unable to retain the important information	2
20. Not enough time is given to allow good communication between health professionals and patients	1
21. Consumers and carers have difficulty understanding key medication information	1
22. Health professionals do not always know how to gauge how much their patients understand^a^	Not applicable^a^

^a^This research priority was inadvertently not visible to participants during the voting activity, and as such, we could not generate a final rank. The A3 poster for this priority topic was placed on the back of a door which was subsequently opened during the workshop, meaning it was hidden from participants’ and facilitators’ view during the voting activity

### Refinement of the priority topics

Four cross-cutting themes emerged from participants’ exploration of the nature of the problem described in each of the 12 priority topics (supported by illustrative quotes from the small group discussion; Fig. 1).

#### Theme 1: Culture and organisational structures

Across the 12 priority topics, participants described how the culture and structures within health services are sometimes insufficient to support good health communication and participation. In this theme, participants talked about issues including professional boundaries and silos, conflict and bullying amongst health professionals, organisational culture that does not value health communication and participation, professional hierarchies that reinforce a lack of consumer control, and poor communication within and across health services.

“*Do doctors feel it’s their job to provide information? Their thinking can be, ‘the next person will do this’.*”

(From priority topic 2)

“*The construction of the concept of quality and safety has an implicit hierarchy, with the consumer at the bottom (i.e. having things done to you by experts).*”

(From priority topic 7)

#### Theme 2: Health professional attitudes and assumptions

In a second theme, discussed in nine priority topics, participants described that healthcare professionals’ assumptions and attitudes towards health communication and participation can underpin the problem. Specifically, this included health professionals making assumptions about consumers’ communication needs, preferences and understanding, and the poor attitudes or discomfort of health professionals to good health communication and participation practices. In three priority topics, participants also said that some health professionals incorrectly assume they already practice good health communication and participation.

“*Assumptions are made by health professionals about the ability of patients to understand information, how much information they want, and their priorities.*”

(From priority topic 2)

“*Health professionals can be reluctant to accept a patient-centred model of care as they think that it means ‘you* [the patient] *will tell me to do things differently’.*”

(From priority topic 1)

#### Theme 3: Inconsistent experiences of care

Across 10 of the priority topics, participants described a variety of issues related to healthcare that is experienced as inconsistent, because it is not personalised to individuals’ and families’ circumstances. Participants shared stories about inconsistent treatment of family and carers, ongoing shortcomings in health services in managing cultural sensitivities, and that it is often an individual consumers’ confidence and ability to be proactive that most determines whether they actively participate in their care. They also shared that health information is not specific enough and too fragmented, and there is too much repetition for patients and family members in the information they must provide in hospital.

“*With my mother whose first language is not English, I am invited into the consultation by the doctors with open arms. But when my husband was in emergency I wasn’t allowed to be in the consultation, I was told to get out.*”

(From priority topic 11)

“*Hospitals tend to pick people* [for consumer advisory committees] *who are not reflective of the diversity of the people they serve. They often go for the low-hanging fruit (retired, white, female and well-educated).*”

(From priority topic 4)

#### Theme 4: Lack of shared understanding in the sector

In the final theme that arose from discussions in seven priority topics, participants described that their priority was underpinned by a lack of shared understanding and common goals between groups and across the sector to inform good health communication and participation. Specific terms and concepts for which a shared understanding or common goals are lacking included patient-centred care, holistic care, quality and safety, health literacy, health communication, and consumer engagement.

“*Health literacy is mostly looked at from professional perspective. What is a consumer perspective of what health literacy means? It’s not often asked.*”

(From priority topic 10)

### Populations and groups for inclusion in future Cochrane Reviews

Participants described a range of different populations and healthcare settings where people are particularly vulnerable to experiencing poor health communication and participation (Fig. 1). In two of the small groups, participants explained that the common thread with many of these groups is the mismatch between consumers’ background (i.e. cultural background, socioeconomic status) and that of their health professionals; the bigger the mismatch, the worse their communication experiences are likely to be.

Participants described at least five different key populations or healthcare settings for each of the 12 priority topics. The most commonly mentioned populations were based on what we termed ‘social health characteristics’, including Indigenous people and people from culturally and linguistically diverse groups, elderly people and people with dementia, people experiencing socioeconomic disadvantage, carers and family members, and young people and those in paediatric care settings. Less frequently mentioned were populations based on what we termed ‘individual health characteristics’, including people being treated in acute care settings, people with rare diseases, people with mental illness and people with chronic disease and multi-morbidity.

### Interventions that could be tested in future Cochrane Reviews

There were 20 different interventions that were suggested as potential foci of future Cochrane Reviews. Given the importance of the intervention to the way in which Cochrane Reviews are framed [34], we provide a complete account of all interventions in Table 4. In each of the small group discussions, participants suggested several different potential interventions (range 5 to 12 interventions suggested per priority topic).

**Table 4:** Interventions that could be tested in future Cochrane Reviews, mapped against the 12 priority topics in which they were described

CCCG intervention taxonomy category [37]	Intervention described by participants	Priority topic number^a^												Total
		1	2	3	4	5	6	7	8	9	10	11	12	
Interventions directed to the consumer	Health information tailored to different audiences and in multiple formats		X			X	X			X	X			5
	Building health literacy skills of consumers						X				X	X		3
	Local and community support interventions					X	X		X					3
	Community education									X				1
Interventions from the consumer	Consumers are partners at all levels of care	X			X			X	X		X			5
	Families and carers, in particular, are partners at all levels of care	X						X	X	X		X		5
	Using patient stories	X	X											2
Interventions for communication exchange between providers and consumers	Patient-controlled electronic health records and related digital tools			X					X	X		X		4
	Communication tools for health professionals	X		X						X			X	4
	Decision aids and decision-making support strategies		X			X								2
	Care plans					X					X			2
Interventions for communication between consumers	Peer-support interventions		X			X	X	X		X		X		6
Interventions for communication to healthcare professionals from another source	Education of health professionals in communication or partnering with consumers	X	X		X	X	X	X		X	X	X	X	10
	Communication skills training for medical students	X								X		X		3
	Strategies to support clinicians having difficult conversations					X				X				2
	Better selection of health professionals							X						1
Service delivery interventions	Health service policies and standards for communication and participation	X		X	X				X	X	X		X	7
	Culture and attitude change within health services and health professionals	X	X	X	X	X				X	X	X		8
	Changes to the structure and delivery of care		X	X			X	X	X				X	6
	Strategies to build on and share good practice within the health system	X			X		X			X		X	X	6
Other	Other (not grouped)					X			X					2

^a^See Table 3 for a description of the 12 priority topics

Considered together, the interventions are multi-directional, covering all six CCCG intervention taxonomy categories, including (1) interventions directed to the consumer (i.e. health information tailored to different audiences and in multiple formats), (2) interventions from the consumer (i.e. consumers are partners at all levels of care), (3) interventions for communication exchange between providers and consumers (i.e. patient-controlled electronic health records and related digital tools), (4) interventions for communication between consumers (i.e. peer support), (5) interventions for communication to healthcare professionals from another source (i.e. education for health professionals in communication or partnering with consumers), and (6) service delivery interventions (i.e. culture and attitude change within health services and health professionals) Fig. 1.

Considered individually, the most commonly described interventions were (1) education for health professionals in communication or partnering with consumers; (2) interventions to change culture and attitude within health services and health professionals; (3) health service policies and standards for good communication and participation; (4) changes to the structure and delivery of care (e.g. nurse-led hospital care or bedside handovers); (5) strategies to build on and share good practice within the health system; and (6) peer support interventions.

### Priority Cochrane Reviews identified

Following the mapping process, application of editorial criteria, and approval from the steering group, CCCG editorial and author teams (as described earlier), the following five Cochrane Reviews were identified as priorities: (1) interventions for improving communication around end-of-life care among health professionals and patients and their families or carers (Henderson, under review); (2) interventions to increase patient and family involvement in escalation of care for acute life-threatening illness in community health and hospital settings [42]; (3) interventions for improving medical students’ interpersonal communication in medical consultations [43]; (4) methods of consumer involvement in developing healthcare policy and research, clinical practice guidelines and patient information material [44]; and (5) interventions for providers to promote a patient-centred approach in clinical consultations [45]. At the time they were selected, three reviews were registered with CCCG as titles [42, 43], and two were existing reviews needing to be updated (that also needed new author teams) [44, 45].

## Discussion

The most highly ranked stakeholder-selected priority topics for health communication and participation systematic reviews were (1) patient-centred care not being well understood or implemented by health services and health professionals; (2) health information provision being of a low priority for health professionals; (3) communication and coordination breakdowns being frequent in health services; and (4) inadequate involvement of consumers in health service planning and design. Four cross-cutting themes described by participants as underpinning the priority topics included organisational culture and structures, health professionals’ assumptions and attitudes, inconsistent experiences of care, and lack of shared understanding in the sector. Key populations for future Cochrane Reviews were more commonly described in terms of social health characteristics, including people from Indigenous or culturally and linguistically diverse backgrounds, elderly people/those with dementia, people experiencing socioeconomic disadvantage, carers/families and young people/those in paediatric settings, than individual health characteristics. A wide range of interventions was suggested for future Cochrane Reviews, most commonly including education for health professionals in communication or partnering with consumers, culture and attitude change within health services and health professionals, and implementing health service policies and standards.

Overall, there was considerable consistency between how the priority topics were ranked in the earlier online survey [26], and those prioritised at the workshop. There were eight priority topics common to those ranked as the top 12 at the workshop and the online survey stage. Notable discrepancies include the priority topics of consumer involvement in research (online survey rank = 1, workshop rank = 18) and ‘official’ health information being contradictory and hard to understand (online survey rank = 2, workshop rank = 15). Both the online survey and workshop participants had a similar focus on equity in terms of populations and a desire for service delivery interventions and training for health professionals. The workshop reinforced these messages and allowed us to probe for further detail about the nature of the priority topics and potential interventions to inform future Cochrane Review selection.

The strengths of this work are that we involved all relevant stakeholder groups, including 50% consumers and carers, and used an explicit, transparent and democratic process to set priority topics supported by a skilled facilitator; these are all strengths recognised in the literature about developing consensus [46]. Such factors are also considered essential ‘process’ components of priority-setting success [47]. We also engaged stakeholders in the additional step of turning the broadly scoped priority topics into specific systematic review questions [22]. By doing so, we offer a method of involving stakeholders in a ‘post-prioritisation’ activity that is typically the sole domain of researchers [48]. Weaknesses include that we did not offer a formal appeals mechanism once the five priority Cochrane Reviews were set [47] and the large-group format used in part A of the workshop may have discouraged contributions from ‘quieter’ participants [49]. Additionally, one A3 poster was inadvertently hidden behind a door during the workshop, meaning that one of the 22 priority topics was not formally voted on. When this was discovered (prior to part B of the workshop) we conducted a ‘show of hands’, to determine if the topic was likely to have been ranked in the top 12. Fewer than seven individuals indicated they wanted to reallocate a sticker to the topic, meaning the results are unlikely to have been affected by this omission.

We are aware of four recent priority-setting exercises conducted with consumers and other stakeholders in topics that overlap with our scope. These include three United Kingdom studies of research priorities in patient safety in primary care [50], ‘fundamental care’ in hospitals [48] and clinical trial recruitment [51], and a United States study of research and practice priorities in patient-centred medication management and adherence [33]. Comparing the results of these studies with ours reveals considerable overlap, and suggests transferability of our results to similar settings. Common research priorities include addressing the health information and support needs of patients and families [33, 48, 50, 51], the provision of person-centred, holistic care tailored to the individual [33, 48, 50], improving communication and coordination between health services and health professionals [48, 50], partnering with consumers (to develop health curricula [33] and in clinical trial planning) [51], and a specific focus on the needs of ‘vulnerable’ groups [50, 51]. Differences include that, in our project, workshop participants had a greater focus on research to solve organisational and cultural barriers to good health communication and participation. This ‘upstream’ focus amongst our stakeholders may reflect the recent introduction of accreditation standards for Australian health services [9] in which they are assessed against a number of quality and safety indicators, including partnering with consumers at all levels of care.

In addition to identifying five priority Cochrane Reviews, this project presents additional opportunities and challenges for CCCG and its review authors. First, stakeholders shone a light on common themes underpinning many health communication and participation challenges in healthcare. They told us that, despite considerable efforts and progress, the power still resides with health professionals and there are often insufficient structures, cultures and practices within health services to support good health communication and participation. The cross-cutting key themes they described may help systematic review authors better understand the context in which their interventions take place, and develop logic models to inform their review [52]. Logic models increasingly feature in systematic reviews of complex interventions, including Cochrane Reviews [53]. We plan to evaluate the implications of being priority reviews with the five teams once they are complete.

Second, our stakeholders want CCCG reviews to consider health equity; those most likely to experience poor health communication and participation should ideally benefit the most from potential interventions. Similarly, stakeholders are not necessarily focussed on health conditions, but predominantly want the reviews that focus on social health characteristics and healthcare settings. One way to address this aspect of health equity would be to conduct reviews that focus specifically on these groups [54]. This is made challenging by the fact that the population of interest in clinical trials is often defined by the health condition, and it is not uncommon for ‘vulnerable’ groups to be excluded from health communication and participation trials (for example, in a Cochrane Review of audio-visual informed consent interventions, 50% of trials excluded people with limited English) [55]. Another approach is to encourage all CCCG authors to explicitly consider equity in the conduct [56] and reporting [54] of their reviews. Participants in this project provided a rich list of potential groups and populations for whom health equity should be considered.

Third, the considerable focus of stakeholders on service delivery interventions raises questions about how best to evaluate the efficacy of system-level interventions in Cochrane Reviews. While the traditional approach within Cochrane Reviews has been to include only ‘rigorous’ non-experimental designs [57], Cochrane contributors are now exploring how ‘diverse data’ (e.g. data from electronic health records, wearable devices and social media) can be incorporated into evidence syntheses [58]. For the CCCG, these diverse data sources could include routinely collected hospital patient experience data [59] and healthcare complaints data [60], but considerable methodological work remains before such data are included in Cochrane Reviews.

Finally, we found that actively involving consumers and other stakeholders to determine priority Cochrane Reviews creates an expectation, and a responsibility, to continue to involve these groups in subsequent review stages. Several workshop participants, steering group members and others expressed a desire to contribute to the priority reviews or to the work of CCCG, more broadly. Given the degree to which the concept of partnering with consumers was a priority for our stakeholders, we had a further mandate to provide ongoing opportunities for involvement. We responded to this by requiring our priority review authors, and any additional reviews led by the CCCG internal editorial team, to actively involve consumers and other stakeholders in their reviews. Cochrane now provides detailed learning materials to support authors to do this [61], but partnering with consumers and other stakeholders is new to many systematic review authors, requires dedicated resources and may extend review timeframes [62].

## Conclusion

Consumers, health professionals and health decision-makers want Cochrane Reviews that address the underlying structural and cultural challenges in health communication and participation, and in doing so explicitly consider health equity. Setting priorities for systematic reviews with consumers and other stakeholders presents a range of additional considerations for systematic review producers.

## Additional files

Additional file 1: Workshop pre-reading pack. (DOCX 410 kb)

Additional file 2: Example of one of the 21 priority topics generated in the earlier online survey stage that was attached to the walls during the workshop. (DOCX 51 kb)

Additional file 3: Workshop agenda. (DOCX 16 kb)

Additional file 4: Small group discussion facilitator template. (DOCX 16 kb)

## Abbreviation

CCCG: Cochrane Consumers and Communication Group
