# Supplementary material for: Selecting, refining and identifying priority Cochrane Reviews in health communication and participation in partnership with consumers and other stakeholders
Source: Health Res Policy Syst. 2019 Apr 29;17:45. doi: 10.1186/s12961-019-0444-z (PMC6489310; doi:10.1186/s12961-019-0444-z)
Supplement: Supplementary file 1 — Workshop pre-reading pack. (DOCX 410 kb) [file 12961_2019_444_MOESM1_ESM.docx]

**Additional file 1 –** Workshop pre-reading pack

Cochrane Consumers and Communication Group

Workshop pre-reading

Setting priorities for health communication and participation research

10 September 2015, Melbourne


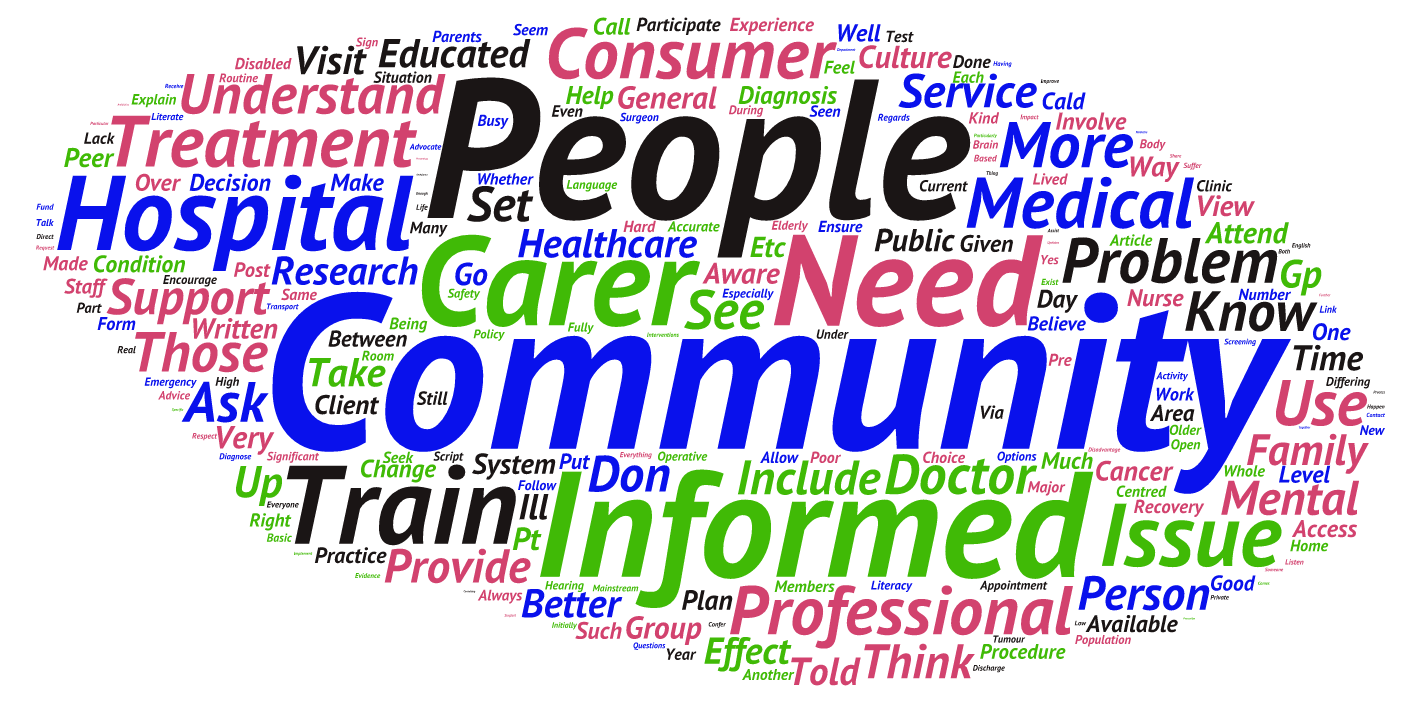


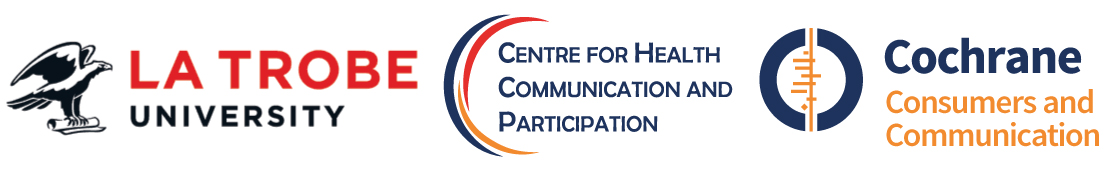


**Thank you for coming along to our workshop!**

**About this pre-reading pack**

We have prepared this pre-reading pack to give you some background information about the project and to share with you what we’ve learnt so far.

We invite you to read this pack before the workshop so you have time to reflect on your own ideas, feedback, suggestions or questions.

We will go through the details here again at the start of the workshop, and will have copies (in colour).

**The key workshop details**

**Date:** Thursday, 10 September

**Time:** 9.45am arrival (for a 10am start) until 4pm

**Venue:** Murdoch Children’s Research Institute (MCRI), Royal Children’s Hospital, Flemington Rd, Parkville (enter via revolving door at front of hospital)

**What we’ll be doing on the day**

In a series of small and large group discussions, you, and 30 other people, will be asked to help us identify a number of priority topics for future research in health communication and participation in health.

The workshop will have three core sessions, with breaks and refreshments in between. The sessions include:

1. Review existing ideas
   - We will present a summary of the suggestions and priorities already collected as part of this project, and seek your reflections on these ideas
2. Generate and rank ideas
   - You will get a chance to identify if there is anything missing, and add new suggestions
   - Then we will ask you to vote on those you think should be the priorities
3. Explore and refine ideas
   - Working in small groups, we will invite you to work on 1 or 2 of the ideas that were voted as high priorities to unpack and refine the topics

**About us and this project**

**About us**

The [Cochrane Consumers and Communication Group](http://cccrg.cochrane.org/) is part of the international organisation, [Cochrane](http://www.cochrane.org/). Cochrane is an independent, not-for-profit organisation dedicated to producing up to date, accurate information about the effects of health care for everyone to access. These assessments of the evidence are known as Cochrane reviews, and they are published (and free to read) on the Cochrane Library (<http://www.cochranelibrary.com/>)

The Consumers and Communication Group publishes reviews of the evidence about interventions, or approaches, which affect the way patients, consumers and carers interact with each other, or with healthcare professionals, health services and health researchers. Specifically, we are interested in activities that promote patients, consumers and carers to be knowledgeable and able to participate in their health in different ways. This includes being able to express their views and beliefs, make informed choices, and minimise risks, and to access high quality health information and health services.

The Cochrane Consumers and Communication Group (<http://cccrg.cochrane.org/>) is based within the [Centre for Health Communication and Participation](http://www.latrobe.edu.au/chcp/) (<http://www.latrobe.edu.au/chcp>) at La Trobe University, Melbourne.

**About this project**

Increasingly, researchers are looking to include consumers, carers, health professionals and policy makers in helping set priorities for research. At the Cochrane Consumers and Communication Group, we want to make sure that we are producing Cochrane reviews on topics that are important to all these groups of people, not just other researchers.

As such, we are conducting a year-long project involving multiple stages. The aims of the project are to:

1. Identify priority topics for systematic reviews of interventions for communication and participation, as judged by Australian and international stakeholders.
2. Identify the top five Australian priorities for new Cochrane reviews within the scope of the Cochrane Consumers and Communication Group.

The first stage involved an online survey to generate suggestions for new research topics, and now we are holding this workshop to refine and prioritise the ideas generated so far. You can read more about the project, and the detailed project outline at <http://www.latrobe.edu.au/chcp/projects/research-priority-setting>

By the end of the 2015, we hope to have five new topics for Cochrane reviews in health communication and participation that have been identified as priorities for Australian consumers, carers, health professionals and policymakers. In 2016, we will be seeking teams of authors to undertake and publish these high priority Cochrane reviews and we will share them widely with Australian and international audiences.

**What we’ve learnt so far: the online survey**

**What we did**

Earlier this year, we invited anyone with an interest in health communication and participation to tell us their ideas for new research topics in this area via an online survey. We promoted the survey to community members, consumer and carer groups, health professionals, health service managers, policy makers, researchers and research funders.

We asked the following questions:

1. What is the health communication and participation problem you would like to see addressed?
2. In your experience, is this a problem for particular groups of people?
3. Is there a particular setting or group of healthcare professionals this is relevant to?
4. Do you have any particular solutions to this problem that you would like to see tested?

**Who responded to the survey?**

Overall, 151 people responded to the survey. Below is some of the key ‘background’ information about the survey respondents:

- Majority Australian (about 75%), other countries were mainly UK, Canada, USA
- Wide range of ages, from 18 to 80 years
- Most were female (about 80%)
- Most were very highly educated (about 85% had a university degree)
- Smaller number of people from non-English speaking backgrounds (about 15%)
- Very small number of Indigenous people (2 people only)
- About one-third (30%) of people identified as a consumer, family/carer or community member
- Nearly half (45%) of people identified as a health professional or health service manager
- Smaller number (15%) of people identified as a researcher, policy maker or research funder
- But most people identified as having more than one ‘perspective’ to contribute (for example, they were a person with a health condition, a family member and a health professional)

**What research priorities did people suggest?**

We received about 200 different topics, problems or suggestions for future research. We sifted and sorted all the responses and grouped the similar ideas together.

Overall, we came up with 21 broad issues, which we grouped together under six different headings. For each issue, we summarised what people said the health communication and participation problem is, who it affects and their possible solutions.

On the next pages is our condensed summary. We will provide more detailed information at the workshop.

In addition, the ‘word cloud’ on the cover page of this pre-reading pack was created from all the most commonly used words in all the survey responses.

**The health communication and participation issues identified**

**The range of issues identified**

Issues related to **health services** more broadly (63 people talked about this)

1. The **quality and safety of patient care** can be compromised by health services (particularly hospitals) not treating patients **holistically**
2. Breakdowns in **communication and coordination** of care between and within health services are common
3. The term **patient-centred care is not well understood** by health services and professionals and poorly implemented
4. **Cultural safety** is not well-embedded in health services
5. **Truly informed consent** for treatment and research does not always happen
6. **Not enough time** to allow good communication between health professionals and patients

Issues related to **health professionals** (34 people talked about this)

1. Some health professionals **don’t understand or ask patients** about their **preferences and priorities**
2. Some health professionals **don’t provide enough information** to patients. Some don’t think it’s a priority for patients.
3. There are often two-way **barriers to adequate communication and participation** (e.g. disability of individual plus discomfort of health professional)
4. Health professionals don’t always provide enough **support for patient decision-making**
5. Health professionals don’t always know how to **gauge how much their patients understand**

Issues for **individual consumers and carers** in their own care (33 people talked about this)

1. Consumers and carers don’t always **know about all the options or services** that exist
2. Patients don’t always **understand their health problems, treatment options or their rights**
3. Consumers and carers aren’t always able to **participate actively** in their care
4. The general public doesn’t always have enough **health literacy** to navigate the health system and make health decisions
5. Patients often experience **information overload** and are unable to retain the important information
6. Consumers and carers have particular issues **understanding key medication** information

Issues for broader **consumer and carer involvement** (31 people talked about this)

1. **Health researchers** don’t adequately **involve patients** in research, nor share their findings
2. Health services don’t properly **involve consumers and carers** in **health service planning and design**

Issues with **publically available information** (18 people talked about this)

1. **‘Official’ health information** can be **contradictory and hard to understand,** both written and online. Consumers and professionals don’t know how **to find and assess good quality** information **online**

Specific issues with **ageing and end of life care** (8 people talked about this)

1. Not enough **support or understanding** about the needs of **older people** and **end of life decisions are poorly understood** by patients, families and the community

**Who these issues affect**

For most of the issues or needs described, people said they affected all patients and/or carers, but that they particularly affected certain groups of people.

These groups included:

- People from culturally and linguistically diverse communities and/or people with limited English
- Carers
- People with limited education/reading/writing skills
- People from low socioeconomic areas
- People with mental health issues
- Older people, including people with dementia/cognitive issues
- People with chronic or multiple illnesses
- People from rural and regional areas
- Aboriginal people
- People with a disability

**Possible solutions suggested**

- Training for health professionals and health services
  - how to better involve patients and carers in their individual care
  - How to communicate with patients and carers, particularly people from culturally and linguistically diverse communities
  - How to involve consumers and carers in the health service more broadly
- Cultural change activities for hospitals and health professionals
- Electronic health records (accessible by patients and carers)
- Support for patients and family members
  - E.g. patient advocates in hospital or peer support workers
- Better information for general public, patients and family members (written and online formats, easy to read, standardised, presents risks and harms)
- Community education campaigns about accessing health services, understanding health
- Training for consumers and researchers in how to involve consumers in research and share research findings in understandable ways
