# Supplementary material for: Selecting, refining and identifying priority Cochrane Reviews in health communication and participation in partnership with consumers and other stakeholders
Source: Health Res Policy Syst. 2019 Apr 29;17:45. doi: 10.1186/s12961-019-0444-z (PMC6489310; doi:10.1186/s12961-019-0444-z)

**Additional file 2 -** Example of one of the 21 priority topics generated in the earlier online survey stage that was attached to the walls during the workshop


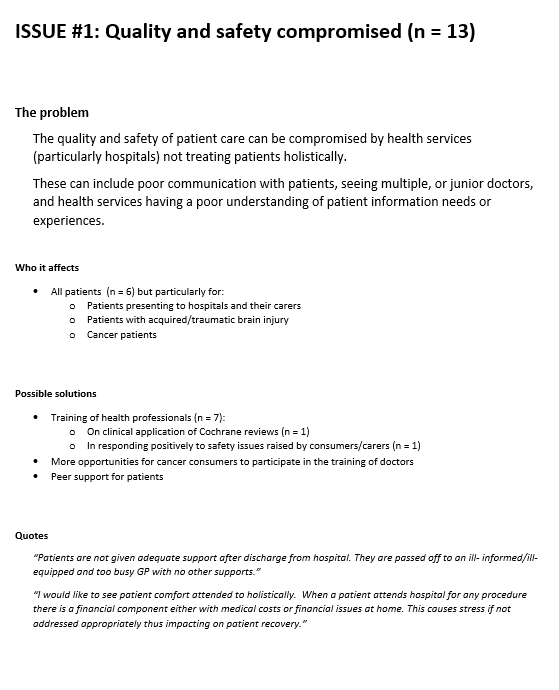

Supplement: Supplementary file 2 — Example of one of the 21 priority topics generated in the earlier online survey stage that was attached to the walls during the workshop. (DOCX 51 kb) [file 12961_2019_444_MOESM2_ESM.docx]
