# Supplementary material for: Selecting, refining and identifying priority Cochrane Reviews in health communication and participation in partnership with consumers and other stakeholders
Source: Health Res Policy Syst. 2019 Apr 29;17:45. doi: 10.1186/s12961-019-0444-z (PMC6489310; doi:10.1186/s12961-019-0444-z)
Supplement: Supplementary file 3 — Workshop agenda. (DOCX 16 kb) [file 12961_2019_444_MOESM3_ESM.docx]

**Additional file 3** – Workshop agenda

| **PRE-WORKSHOP** | |
| --- | --- |
| 9.45-10.00 (15 mins) | **Arrival**  Coffee and tea on arrival |
| **SESSION 1: REVIEW OF IDEAS TO DATE** | |
| 10.00-11.15  (75 mins) | 1. Brief introductions 2. Recap on scope and purpose, plan for the day 3. Presentation of what we’ve learnt so far |
| 11.15-11.30  (15 mins) | Morning tea |
| **SESSION 2: GENERATE AND RANK IDEAS** | |
| 11.30-12.15  (45 mins) | 1. Reflections on ideas generated so far 2. Discussions about what could be added |
| 12.15-1.00  (45 mins) | 1. Voting on issues around the room |
| 1.00-1.45 (45 mins) | Lunch |
| **SESSION 3: EXPLORE AND REFINE IDEAS** | |
| 1.45 – 3.00  (75 mins) | Explore and refine the key features of the high-ranking issues in small groups |
| 3.00-3.15 | Afternoon tea |
| 3.15-4.00  (45 mins) | Feedback forms  Brief report back from small groups  Wrap up, thank yous, next steps |
| **CLOSE** | |
