# Supplementary material for: Selecting, refining and identifying priority Cochrane Reviews in health communication and participation in partnership with consumers and other stakeholders
Source: Health Res Policy Syst. 2019 Apr 29;17:45. doi: 10.1186/s12961-019-0444-z (PMC6489310; doi:10.1186/s12961-019-0444-z)
Supplement: Supplementary file 4 — Small group discussion facilitator template. (DOCX 16 kb) [file 12961_2019_444_MOESM4_ESM.docx]

**Additional file 4** - small group discussion facilitator template

**Facilitator name:___________________________**

**Issue/problem discussed with small group:______________________________________________**

1. **Invite free-flowing discussion about people’s thoughts or reflections on the issue identified**

Does everyone on the table agree it’s a high priority issue?

How does this relate to their own experiences?

Are there concepts or ideas that are missing?

Make a note of the key ideas discussed

|  |
| --- |

1. **Explore the description of the problem in more detail**

Does the description of the problem cover everything?

Are there other things worth mentioning?

Are there any specific sectors or parts of the health system where this is worse?

*Note for facilitators: this will help us understand the context/background/justification for a potential Cochrane review*

|  |
| --- |

1. **Explore who it affects in more detail**

Who and how does this affect people?

Which groups of people are worse off or may find it harder to manage?

Any other features, eg younger or older etc?

*Note for facilitators: here we are trying to clarify the P in the PICO*

|  |
| --- |

1. **Explore the potential solutions in more detail**

Have you seen, heard or read about any ways of tacking this problem?

Would you like to know if any specific strategies have worked?

*Note for facilitators: here we are trying to clarify the I in the PICO)*

|  |
| --- |
